# Involvement of Heat Shock Proteins on the Transcriptional Regulation of Corticotropin-Releasing Hormone in Medaka

**DOI:** 10.3389/fendo.2019.00529

**Published:** 2019-08-02

**Authors:** Tomoya Uchimura, Seiji Hara, Takashi Yazawa, Yasuhiro Kamei, Takeshi Kitano

**Affiliations:** ^1^Department of Biological Sciences, Graduate School of Science and Technology, Kumamoto University, Kumamoto, Japan; ^2^Department of Biochemistry, Asahikawa Medical University, Asahikawa, Japan; ^3^Spectrography and Bioimaging Facility, National Institute for Basic Biology Core Research Facilities, National Institute for Basic Biology, Okazaki, Japan

**Keywords:** corticotropin-releasing hormone, cortisol, heat shock protein, hypothalamus, medaka, sex determination

## Abstract

Medaka (*Oryzias latipes*) are teleost fish with a XX/XY sex determination system. Recently, it was reported that high temperature (HT) induced the masculinization of XX medaka by increasing the levels of cortisol, a major glucocorticoid produced by interrenal cells in teleosts. Cortisol secretion is regulated by adrenocorticotropic hormone (ACTH) secreted from the pituitary gland, which is partly regulated by corticotropin-releasing hormone (CRH) secreted from the hypothalamus. In teleosts, two *crh* paralogs, named *crha* and *crhb*, have been identified. Recently, the expression of *crhb* but not *crha* was upregulated by HT during gonadal sex differentiation period in medaka and loss-of-functions of its receptors under HT suppressed masculinization of XX medaka and increase of cortisol levels, suggesting that *crhb* is involved in masculinization induced by HT. However, the transcriptional regulation of *crhb* under HT has not been elucidated. We analyzed the gene expression pattern in the hypothalamus of medaka embryos incubated under HT using DNA microarray. The expressions of heat shock protein (hsp) genes, such as *hsp70.1* and *hsp30*, were increased. Overexpression of *hsp70.1* or *hsp30* in cultured rat hypothalamic 4B cells significantly induced *crh* gene expression. Moreover, hypothalamic *hsp70.1*-overexpressing transgenic medaka also showed increased *crhb* gene expression that increased cortisol levels compared with fish incubated at a normal temperature. These results provide the first evidence that HSPs induce cortisol levels by elevating *crhb* gene expression in the hypothalamus.

## Introduction

In vertebrates, sex is determined primarily by a combination of sex chromosomes at fertilization. However, the mechanism of sex determination in vertebrates is diverse, and environmental stresses such as temperature and individual interaction affect sex determination in vertebrates other than mammals (1). The elucidation of sex determination and sexual differentiation mechanisms is expected to be the basis of applied research for the preservation of biological species and reproductive medicine.

Medaka (*Oryzias latipes*) are small fish with several desirable features for use as a model organism, including a short generation time, small genome size, and the availability of numerous useful strains (2). Transgenic (Tg) techniques and gene knockout system using the clustered regularly interspaced short palindromic repeats (CRISPR)/CRISPR-associated protein 9 (Cas9), have already been established in this fish (3–5). Furthermore, the medaka sex-determining gene *dmy/dmrt1bY*, which is found on the Y chromosome, has been identified (6–8). Thus, medaka provides an excellent vertebrate model for investigating various biological phenomena including embryonic development and sex differentiation. Recently, it was reported in medaka that exposure to water of a high temperature (HT) during the sex differentiation period induced female-to-male sex reversal (9, 10). Additionally, HT caused the masculinization of XX medaka by elevating the levels of cortisol (11, 12), the major glucocorticoid produced by the interrenal cells in teleosts (13). However, it remains unclear how HT-elevated cortisol induces the masculinization of XX fish.

Cortisol is a glucocorticoid produced in the adrenal cortex that regulates the expression of various stress response genes via a glucocorticoid receptor (13). In addition, cortisol secretion is regulated by adrenocorticotropic hormone (ACTH) secreted from the pituitary gland, and which is partly regulated by corticotropin-releasing hormone (CRH) secreted from the hypothalamus (14). In teleosts, two *crh* paralogs, named *crha* and *crhb*, have been identified (15). The expressions of *crha* and *crhb* have been mainly observed in the retina and brain, respectively (15, 16). Recently, the expression of *crhb* but not *crha* was upregulated by HT during gonadal sex differentiation period in medaka and loss-of-functions of its receptors under HT suppressed masculinization of XX medaka and increase of cortisol levels, suggesting that *crhb* is involved in masculinization induced by HT (17). However, the transcriptional regulation of *crhb* under HT has not been elucidated.

Here, we performed DNA microarray analysis using the hypothalamic regions of medaka embryos reared under HT to identify novel factors regulating *crh* expression. We confirmed an increase in the gene expressions of heat shock proteins (hsps) such as *hsp70* and *hsp30*. HSPs function as molecular chaperones under physical (18) and environmental stress to maintain homeostasis of the higher order structures of various proteins (19). We performed a functional analysis of HSPs on the transcriptional regulation of *crh* using cultured rat hypothalamus cells and medaka brains.

## Materials and Methods

### Animals

The FLFII medaka stock was used (20), which allows the identification of genotypic sex by the appearance of leucophores at 2 days post-fertilization (dpf), before the onset of sex differentiation. The *neurogenin3* (*ngn3)-green fluorescent protein (GFP)* Tg medaka line was generated by injecting the pEGFP-1 vector (Clontech, Palo Alto, CA) fused to the regulatory region of medaka *ngn3*, which is expressed strongly in the hypothalamus (21), into one-cell stage embryos of FLFII stock as previously described (22). All injected embryos were bred to adults and only F1 embryos possessing GFP fluorescence were selected and used to produce succeeding generations.

To generate *hsp70.1*-overexpressing Tg medaka that overexpressed HSP70.1 in the hypothalamus, both *ngn3-hsp70.1*, where full-length medaka *hsp70.1* cDNA (23) was exchanged with *EGFP* cDNA in an *ngn3-GFP* plasmid, and the *olvas-DsRedExpress* plasmid, which is used to visualize germ cells by DsRed fluorescence (11), were injected into one-cell stage embryos of FLFII stock (Supplementary Figure 1). All embryos were bred to adults and mated with each other to obtain heterozygous Tg medaka (F1). Only embryos possessing DsRed fluorescence were selected and homozygous Tg medaka produced by mating F1 fish with each other were used to perform the experiments.

Medaka embryos and larvae were maintained in ERM (17 mM NaCl, 0.4 mM KCl, 0.27 mM CaCl_2_.2H_2_O, 0.66 mM MgSO_4_, pH 7) at 26°C (normal temperature) or 33°C (HT) under a 14-h light and 10-h dark cycle. Fish images were captured using a BZ-9000 BioRevo fluorescence microscope (Keyence Co., Osaka, Japan). Survival rates were investigated by counting individual numbers with a heartbeat. Developmental stages of the embryos were determined as described previously (24).

### Immunohistochemistry

The *ngn3-GFP* Tg embryos at 4 dpf (stage 33) and adults at about 6 months of age were fixed in Bouin's solution at 4°C overnight, dehydrated in graded ethanol, embedded in paraffin, and sectioned serially at 5 μm as previously described (25). After deparaffinization and rehydration, sections were incubated at 4°C overnight with primary antibodies (1:500 dilution), mouse monoclonal anti-GFP (Roche Diagnostics, Indianapolis, IN). After washing, the sections were treated for 1 h with secondary antibodies (1:500 dilution), anti-mouse IgG conjugated with biotin (Molecular Probes, Eugene, OR). Then, they were developed using a Histofine SAB-PO kit (Nichirei Co., Tokyo, Japan) and counterstained with hematoxylin.

### DNA Microarray Analysis

Total RNA was extracted from the GFP-positive hypothalamic regions cut off from *ngn3-GFP* Tg embryos (50 pooled fishes) incubated at normal temperature (control) or HT at 4-dpf, before the onset of the elevation in cortisol levels (11), using RNeasy Mini Kit (Qiagen, Hilden, Germany). The DNA microarray analysis was conducted as shown in our previous paper (26). Briefly, DNA microarray hybridization and analyses following the protocol of the manufacturer for one color, microarray-based gene expression analysis (version 5.5) were performed with a medaka microarray tip (44 K; Agilent Technologies, Santa Clara, CA) in the Chemical Evaluation and Research Institute (Tokyo, Japan). Based on Agilent Feature Extraction 12.0, the median of the selected raw data was calculated for each array, and normalization to the median was then applied to the raw intensities across the arrays. Differentially expressed genes from the HT group were selected by *t*-tests with *P*-values <0.05 and based on a change that was 2-fold greater than that of the control group. The up-regulated or down-regulated genes were annotated by biological process and cellular component based on Gene Ontology (GO) using the GO consortium (http://www.geneontology.org/) and UniProtKB/Swiss-Prot (http://www.uniprot.org/).

### Cell Culture

Rat hypothalamic 4B cells were cultured in Dulbecco's modified eagle's medium (DMEM; Invitrogen Co., Carlsbad, CA) supplemented with 10% charcoal/dextran-treated fetal bovine serum (CDFBS; HyClone, Logan, UT), 100 mg/ml streptomycin, and 100 U/ml penicillin at 37°C in 5% CO_2_ and passaged based as previously described (27). The cells were cultured in the medium for 2 h at 37 or 45°C with or without 1 × 10^−5^ M HSP inhibitor (KNK 437, Calbiochem). KNK437 inhibited heat shock factor activity and heat-induced *hsp* expression in human and *Xenopus* cultured cells (28, 29). After treatment, the cells were collected, and total RNA was extracted for the analysis of gene expression.

### Constructs and Transfection

The *hsp70.1*- and *hsp30*-expression plasmids were constructed by cloning the full-length medaka *hsp70.1* and *hsp30* cDNAs into pcDNA3.1 (Invitrogen). 4B cells were plated in 24-well plates 24 h before transfection with 100 ng of the *hsp70.1*- or *hsp30*-expression plasmids, or an empty plasmid (control) using Lipofectamine reagent (Invitrogen) as previously described (25). After 24 h transfection, the cells were collected, and total RNA was extracted for the analysis of gene expression.

### Organ Culture

Brains dissected from *hsp70.1*-overexpressing Tg and wild-type XX medaka embryos (4 dpf) incubated at 26°C were used for organ culture. The brains were cultured in Leibovitz's L-15 medium (Invitrogen) for 16 h at 26 or 33°C with or without 1 × 10^−5^ M HSP70 functional inhibitor (PES: 2-phenylethynesulfonamide, Calbiochem). PES is a functional inhibitor that selectively acts on HSP70 to inhibit the association between HSP70 and its co-chaperone (30). After culturing, the brains were used for the analysis of gene expression.

### Quantitative Reverse Transcription-Polymerase Chain Reaction (qRT-PCR)

Total RNA was extracted from medaka tissues and cultured rat cells using ISOGEN (Nippon Gene, Tokyo, Japan). Then, 0.2 μg of the extracted RNA was reverse-transcribed using an RNA PCR kit (Applied Biosystems) at 42°C for 30 min. Quantitative real-time PCR was performed in a LightCycler 480 (Roche Diagnostics GmbH, Mannheim, Germany) using SYBR Green I Master (Roche) as previously described (12). Primers used are shown in Supplementary Table 1. The conditions were as follows: preheating PCR was carried out at 95°C for 5 min, 95°C for 10 s, 59°C for 10 s, and 72°C for 10 s for 45 cycles. Evaluation of the copy number of the target genes was calculated based on *ef1*α in medaka cells or *b2mg* in rat cells. All experiments were performed in triplicates.

### Measurement of Cortisol

Steroid hormones in XX medaka larvae (5 pooled fishes) at 0 days post-hatching (dph) (stage 39) were extracted with diethyl ether as previously described (11, 31). Cortisol levels were measured using a Cortisol EIA kit (Cayman Chemical, Ann Arbor, MI) in accordance with the manufacturer's instructions.

### Statistical Analysis

Experimental results were tested using Levene's test for homogeneity of variance. Data were analyzed by the Student's *t*-test or one-way ANOVA followed by Tukey's multiple comparison test using SPSS statistics 20 (IBM Corp., Armonk, NY).

## Results

### Generation of a Tg Medaka Line to Visualize the Hypothalamus by GFP Fluorescence

**Figure 1:**
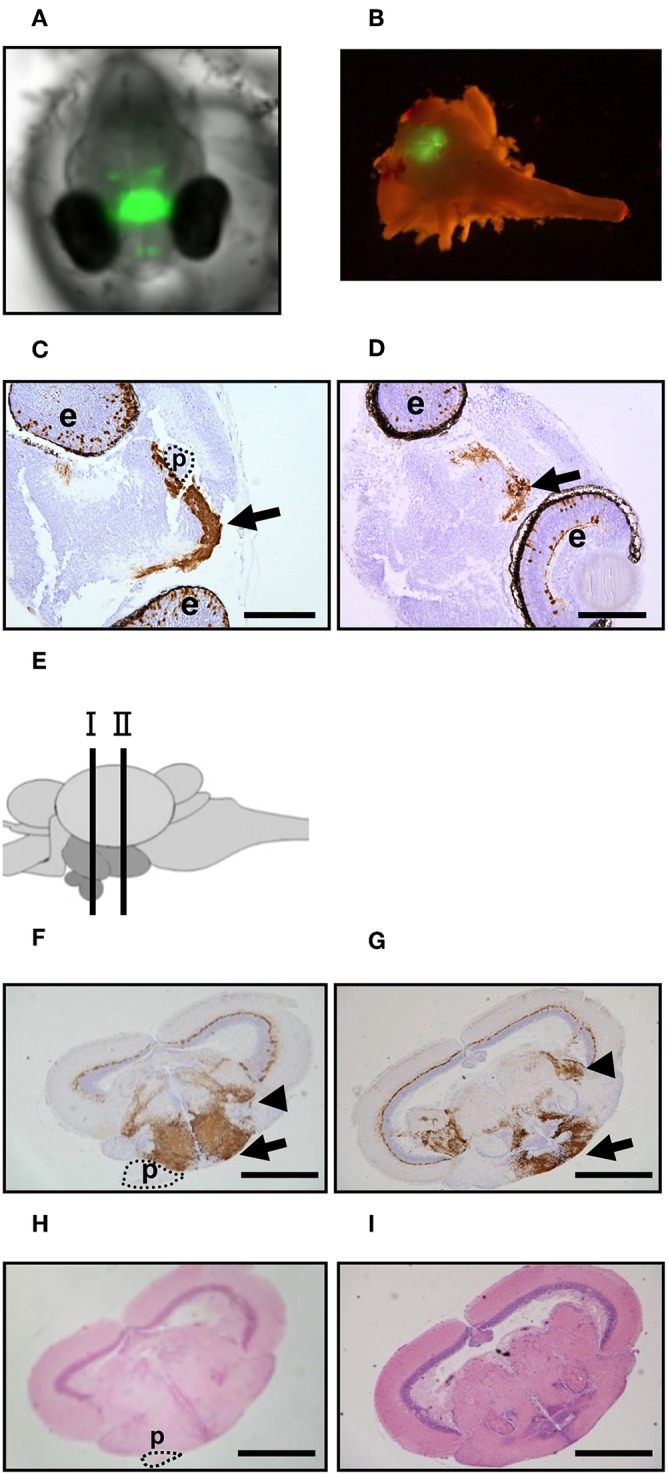
GFP expression in *ngn3-GFP* Tg medaka. **(A,B)** GFP fluorescence in the Tg XX medaka at 4 dpf **(A)** and adult brain **(B)**. **(C,D)** Immunohistochemistry to detect GFP was performed using head tissues of the embryos treated at normal temperature **(C)** or high temperature **(D)**. Arrow: hypothalamus. Scale bar: 100 μm. e, eye; p, pituitary. **(E)** Schematic illustration of the section sites in adult medaka brains. Site I contain the pituitary and site II does not contain the pituitary. **(F–I)** Immunohistochemistry to detect GFP **(F,G)** and HE staining **(H,I)** were performed using brain tissues of the adults reared at normal temperature. **(F,H)** Site I and **(G,I)** Site II. Arrow: hypothalamus. Arrowhead: area pretectalis. Scale bar: 500 μm. p, pituitary.

To visualize the hypothalamus in medaka embryos, we generated a *ngn3-GFP* Tg medaka line by injecting a *pEGFP-1* vector fused to the regulatory region of medaka *ngn3*, which is expressed strongly in the hypothalamus (21), into the fertilized eggs of FLFII stock (Figures 1A,B). Immunohistochemical staining using anti-GFP antibody showed positive signals were detected mainly in the eyes and hypothalamus of embryos incubated at 26°C (control; Figure 1C) and 33°C (HT; Figure 1D). Moreover, immunohistochemical staining of the adult brain showed signals were detected strongly in the hypothalamus containing the nucleus ventral tuberis (Figures 1E–I), where medaka *crha* and *crhb* mRNAs are co-expressed (16), the area pretectalis and the ventral part of the optic tectum. Thus, this Tg line allows visualization of the hypothalamus by GFP fluorescence in living individuals.

### Analysis of Gene Expression Changes in Medaka Embryos by HT

**Figure 2:**
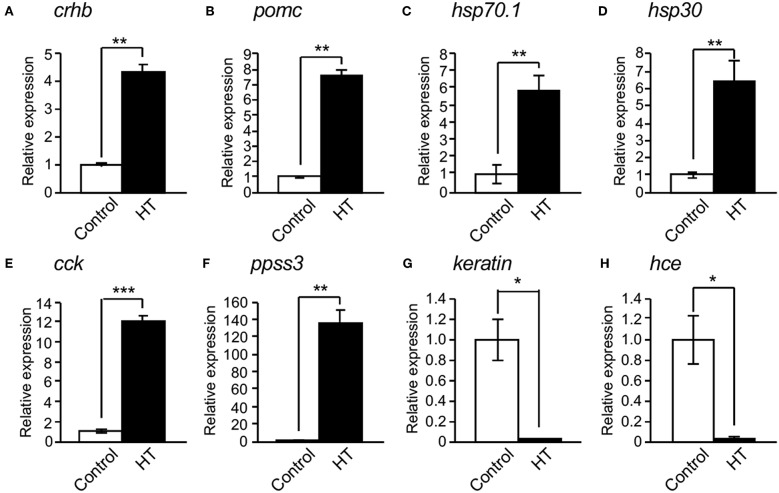
Confirmation of mRNA expression changed by high temperature. **(A–F)** qRT-PCR showed that high temperature (HT) increased the expressions of *crhb* **(A)**, *pomc* **(B)**, *hsp70.1* **(C)**, *hsp30* **(D)**, *cck* **(E)**, and *ppss3* mRNAs **(F)** in the hypothalamic region of XX embryos at 4 dpf. **(G,H)** qRT-PCR showed that HT decreased the expressions of *keratin* **(G)** and *hce* mRNAs **(H)** in the hypothalamic region of XX embryos at 4 dpf. Vertical bar: mean ± standard error of triplicates, ^*^p < 0.05, ^**^p < 0.01, ^***^p < 0.001.

To identify novel factors regulating CRH expression in the hypothalamus, we performed DNA microarray analysis using the hypothalamic regions (containing the pituitary) of medaka embryos reared at 26 and 33°C. HT treatment induced the expressions of 2,512 genes more than 2-fold in 44,000 genes, of which 65 genes had expressions increased more than 10-fold (Supplementary Figure 2). These 65 genes included *hsp70.1, hsp30, cholecystokinin (cck)*, and *somatostatin 3 (ppss3)*. HT treatment reduced the expression of 1,981 genes <1/2, of which 58 genes were reduced <1/10 (Supplementary Figure 2). These 58 genes included *keratin*, and *choriolysin H (hce)*. Furthermore, qRT-PCR analysis of the hypothalamus regions of medaka embryos at 4 dpf confirmed that *crhb, proopiomelanocortin (pomc), hsp70.1, hsp30, cck*, and *ppss3* expressions were significantly induced by HT (Figures 2A–F), while *keratin* and *hce* expressions were significantly reduced by HT (Figures 2G,H), similar to the DNA microarray analysis.

### Effects of HSPs on *crh* Expression in Cultured Hypothalamic Cells

**Figure 3:**
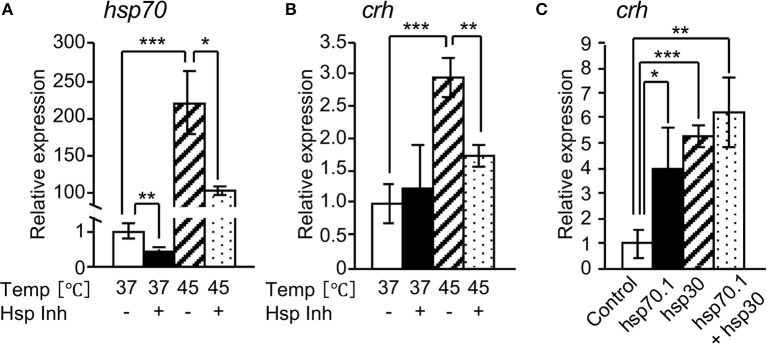
Effects of HSPs on *crh* expression in 4B cells. qRT-PCR shows that HSP inhibitor (Hsp Inh) significantly attenuated the expressions of *hsp70* **(A)** and *crh* mRNAs **(B)**. **(C)** qRT-PCR showed that the forced expression of *hsp70.1* and/or *hsp30* by transfection of an expression vector significantly induced the expression of *crh* mRNA. Vertical bar: mean ± standard error of triplicates, ^*^p < 0.05, ^**^p < 0.01, ^***^p < 0.001.

We investigated the effect of HSPs on *crh* expression in rat hypothalamus-derived 4B cells. *hsp70* and *crh* expressions were markedly increased by heat shock (45°C) of the cells, and their expressions were significantly inhibited by treatment with a HSP inhibitor (KNK 437) under heat shock conditions (Figures 3A,B). *crh* expression was significantly induced by the overexpression of *hsp70.1* or *hsp30* in 4B cells, and was further induced by their co-overexpression (Figure 3C).

### Analysis of Hypothalamic *Hsp70.1*-Overexpressing Tg Medaka

**Figure 4:**
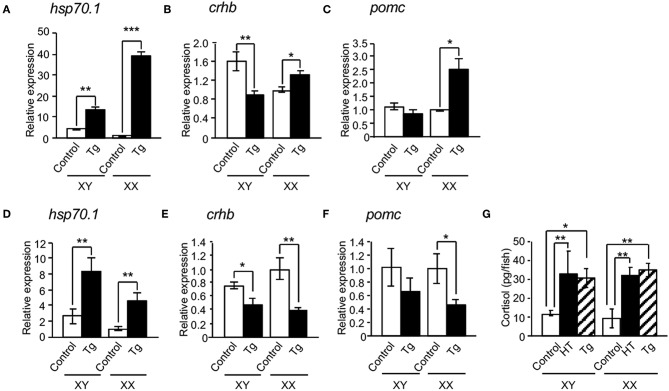
Effects of HSP70 on the expressions of cortisol-regulated genes in *hsp70.1*-overexpressing Tg medaka. In the hypothalamic regions of XX medaka at 4 dpf **(A–C)** and 0 dph **(D–F)**, the expressions of *hsp70.1* **(A,D)**, *crhb* **(B,E)**, and *pomc* mRNAs **(C,F)** were investigated by qRT-PCR. **(G)** Whole-body levels of cortisol in the larvae at 0 dph. HT: high temperature treatment, Tg: *hsp70.1*-overexpressing Tg fish. Vertical bar: mean ± standard error of triplicates, ^*^p < 0.05, ^**^p < 0.01, ^***^p < 0.001.

We investigated the effects of HSP70.1 on the expressions of cortisol-regulated genes in hypothalamic *hsp70.1*-overexpressing Tg medaka. qRT-PCR showed that the expression of *hsp70.1* mRNA was significantly increased in the hypothalamic regions of both sexes of Tg larvae at 4 dpf and 0 dph compared with controls (Figures 4A,D), while all expressions of *crhb* and *pomc* mRNAs were not increased in the hypothalamic regions of the larvae at 4 dpf and 0 dph compared with controls (Figures 4B,C,E,F). Whole-body levels of cortisol in both sexes of Tg larvae at 0 dph were significantly elevated compared with controls, similar to those in HT-treated larvae (Figure 4G). Survival rates in Tg medaka incubated at 26°C were 100% over 5 days, similar to those in controls, whereas they were higher in Tg fish incubated at 33°C than in controls incubated at 33°C (Supplementary Table 2).

**Figure 5:**
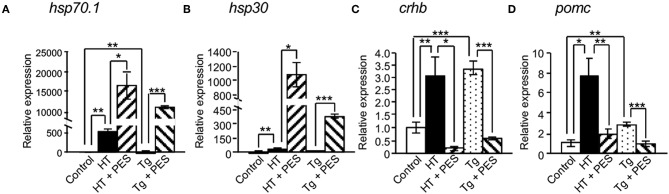
Effects of HSP70 on the expressions of cortisol-regulated genes in medaka brains treated with or without an HSP70 functional inhibitor (PES). The expressions of *hsp70.1* **(A)**, *hsp30* **(B)**, *crhb* **(C)**, and *pomc* mRNAs **(D)** in brains of XX embryos at 4 dpf were investigated by qRT-PCR. HT: high temperature treatment, Tg: *hsp70.1*-overexpressing Tg fish. Vertical bar: mean ± standard error of triplicates, ^*^p < 0.05, ^**^p < 0.01, ^***^p < 0.001.

To bypass the effects of the negative feedback system through the hypothalamic-pituitary-adrenal (HPA) axis in *hsp70.1*-overexpressing Tg medaka, we performed organ culture experiments using brains dissected from embryos at 4 dpf. qRT-PCR showed that the expressions of *hsp70.1, crhb* and *pomc* mRNAs were significantly increased in the brains of Tg embryos compared with controls (Figures 5A,C,D), while the expressions of *crhb* and *pomc* mRNAs were significantly inhibited by treatment with an HSP70 functional inhibitor (PES), similar to HT-treated embryos (Figures 5C,D). PES treatment increased the expressions of *hsp70.1* and *hsp30* mRNAs in the brains of Tg embryos similar to HT-treated embryos (Figures 5A,B).

## Discussion

In this study, we identified novel factors regulating CRH expression in the hypothalamus by DNA microarray analysis using the hypothalamic regions of *ngn3-GFP* Tg medaka embryos reared at normal temperature and HT. Sixty-five genes (including *hsp70.1* and *hsp30*) had expression levels increased more than 10-fold in medaka reared at HT compared with those reared at normal temperature. HSPs function as molecular chaperones under physical (18) and environmental stress to maintain homeostasis of the higher order structures of various proteins (19). Recently, it was reported that some HSPs in medaka were regulated by heat shock factor 1, which is activated by heat stress (32), suggesting HSPs may have common regulating mechanisms in vertebrates. Therefore, we focused on the effects of HSPs on *crh* expression.

We investigated the effect of HSPs on *crh* expression in rat hypothalamus-derived 4B cells. *hsp70.1* and *crh* expressions were markedly elevated by heat shock, and significantly inhibited by treatment with an HSP inhibitor under heat shock. This suggested that change in the level of HSPs modulate the *crh* expression in hypothalamic cells. Moreover, *crh* expression was significantly induced by the overexpression of *hsp70.1* or *hsp30* in 4B cells, and further increased by their co-overexpression. Therefore, *hsp70.1* and *hsp30* are likely to have capacity for inducing *crh* expression in hypothalamic cells.

Next, we investigated the effect of HSP70.1 on the expression of cortisol-regulated genes in medaka hypothalamic cells using hypothalamic *hsp70.1*-overexpressing Tg medaka. qRT-PCR showed that the expression of *hsp70.1* mRNA was significantly increased in hypothalamic regions of Tg larvae compared with controls, while the expressions of *crhb* and *pomc* mRNAs were decreased in hypothalamic regions of the larvae. This indicated the existence of a negative feedback system mediated through the HPA axis (33). To bypass the effects of this system through the HPA axis in Tg medaka, we performed organ culture experiments using embryo brains. qRT-PCR showed that the expressions of *hsp70.1, crhb*, and *pomc* mRNAs were significantly increased in the brains of Tg embryos compared with controls, while the expressions of *crhb* and *pomc* mRNAs were significantly inhibited by treatment with an HSP70 functional inhibitor, similar to those in HT-treated embryos. These results strongly suggest that HSP70.1 induces *crhb* expression in the medaka hypothalamus. The human CRH gene contains a cyclic AMP-responsive element (CRE) and several partial 12-O-tetradecanoyl phorbol 13-acetate (TPA) response elements (TREs) (34). Numerous studies have suggested that protein kinase A (PKA) and protein kinase C (PKC) stimulators increase the expression of CRH. Moreover, HSP70 binds to the carboxyl terminus of at least three AGC kinase family members: PKC isozymes, Akt, and PKA (35). This interaction is mediated primarily by the peptide-binding domain on HSP70 and the dephosphorylated turn motif on protein kinases (35), suggesting that HSP70 activates these kinases. Although it remains unclear whether CRE and TREs are present in the medaka *crhb* gene promoter, HSP70 may regulate *crhb* expression through the activation of these kinases in vertebrates.

Previously, restraint stress which activates the HPA axis, has induced *hsp70* expression by elevating ACTH levels in rat adrenal cortex (36). Long-term dexamethasone treatment has reduced adrenal restraint-induced *hsp70* expression (37), suggesting a functional interrelationship between HPA activity and *hsp70* expression in the adrenals. Moreover, it was reported that ubiquitously HSP70-overexpressing Tg mice suppressed apoptosis and had increased levels of serum corticosterone (38). In this study, whole-body levels of cortisol in the hypothalamic *hsp70.1*-overexpressing Tg larvae at 0 dph were significantly elevated compared with controls, similar to those in HT-treated larvae. Therefore, HSP70 appears to activate the HPA axis via *crh* expression in common among vertebrates.

We previously showed that HT inhibited the female-type proliferation of germ cells and induced the masculinization of XX medaka by elevating the levels of cortisol (11). Preliminary results showed that although this *hsp70.1*-overexpressing Tg adults did not undergo female-to-male sex reversal, germ cell proliferation in Tg XX larvae at 0 dph was partially inhibited compared with wild-type XX fish (data not shown), suggesting that overexpression of HSP70.1 in the hypothalamus may inhibit the female-type proliferation of germ cells by increasing the levels of cortisol. Taken together, these results strongly suggest that hypothalamic HSP70.1 induced by heat stress inhibits the female-type proliferation of germ cells to induce masculinization of XX medaka by increasing the levels of cortisol. Future studies should focus on the phenotypic analysis of this Tg medaka.

In summary, DNA microarray analysis showed that the expressions of *hsp* genes, such as *hsp70.1* and *hsp30*, were increased in the hypothalamus of medaka embryos incubated under HT compared with those at normal temperature. The overexpression of *hsp70.1* or *hsp30* in cultured rat hypothalamic 4B cells significantly induced *crh* gene expression. Moreover, *hsp70.1*-overexpressing Tg medaka had elevated *crhb* gene expression and increased cortisol levels compared with medaka incubated at normal temperature. These results provide the first evidence that HSPs induce cortisol levels by elevating *crh* gene expression in the hypothalamus.

## Data Availability

The datasets generated for this study can be found in the Supplementary Table 1.

## Ethics Statement

The animal study was reviewed and approved by Kumamoto University. Written informed consent was obtained from the owners for the participation of their animals in this study.

## Author Contributions

TK and YK obtained funding and designed the study. TU, SH, TY, YK, and TK performed the experiments and collected the data. TU and TK wrote the manuscript.

### Conflict of Interest Statement

The authors declare that the research was conducted in the absence of any commercial or financial relationships that could be construed as a potential conflict of interest.

## Abbreviations

HT: high temperature
ACTH: adrenocorticotropic hormone
CRH: corticotropin-releasing hormone
HPA: hypothalamic-pituitary-adrenal
HSP: heat shock protein
qRT-PCR: quantitative reverse transcription-polymerase chain reaction
GFP: green fluorescent protein.
