# Supplementary material for: Involvement of Heat Shock Proteins on the Transcriptional Regulation of Corticotropin-Releasing Hormone in Medaka
Source: Front Endocrinol (Lausanne). 2019 Aug 2;10:529. doi: 10.3389/fendo.2019.00529 (PMC6688511; doi:10.3389/fendo.2019.00529)
Supplement: Supplementary file 1 [file Table_1.pdf]

Supplementary Table 1. List of primers used in this study.

Gene	Accession No.	Primer sequences
hsp70(rat)	L16764	For, 5'-ATGGCCAAGAAAACAGCGATC<br>Rev, 5'- CCAGTGCTTCATGTCCGACT
crh(rat)	M54987	For, 5'- TGGATCTCACCTTCCACCTTCTG<br>Rev, 5'- CCGATAATCTCCATCAGTTTCCTG
b2mg(rat)	Y00441	For, 5'- CGGTGACCGTGATCTTTCTGGT<br>Rev, 5'- GGTGACGGTTTTTGGGCTCCTT
hsp70.1(medaka)	AF286875	For, 5'- AAGCCGAGGACGAGCAGCA<br>Rev, 5'-TTGCAGACCTTCTCCAGCTC
hsp30(medaka)	CP020676	For, 5'- CGCTACTCTTACAGACTCC<br>Rev, 5'-GATGTATTGGGAGTCTGGAC
ppss3 (medaka)	CP020671	For, 5'- GTTCTGTTCAGCGTTAGAGC<br>Rev, 5'- GATAACCTGTTATAGACAGC
cck(medaka)	CP020675	For, 5'- CTCAGCTCCTTCTGAAGTTG<br>Rev, 5'- ACGCCTCCTCTCTGTTGTTTGC
keratin(medaka)	DE262775	For, 5'- CAGTCGTCTCCTCACAACTTC<br>Rev, 5'- TACGAATATCTCCAGGTCAC
hce(medaka)	083950	For, 5'-AGCTCTTGCTAGAAGGAGAC<br>Rev, 5'-ATAGACAGCTCCTGTAGTCC
crhb(medaka)	AB070612	For, 5'- TGGTACCACCGTCATTCTGC<br>Rev, 5'- AAGCCGCTTAGAAGCGCTCT
pomc(medaka)	CP020666	For, 5'- CCTCCTCATCTCACCTGGGA<br>Rev, 5'- GAGAGCAGGATGACGGAGTG
ef1a(medaka)	AB013606	For, 5'- TGAGATGGGCAAGGGCTCCT<br>Rev, 5'-GCTGGGTTGTAGCCGATCTT
