# Supplementary material for: Involvement of Heat Shock Proteins on the Transcriptional Regulation of Corticotropin-Releasing Hormone in Medaka
Source: Front Endocrinol (Lausanne). 2019 Aug 2;10:529. doi: 10.3389/fendo.2019.00529 (PMC6688511; doi:10.3389/fendo.2019.00529)
Supplement: Supplementary file 2 [file Table_2.pdf]

Supplementary Table 2. Changes in survival rates.

After treated		1 day	2 day	3 day	4 day	5 day	TOTAL
26℃	Control	100%	100%	100%	100%	100%	21
	Tg	100%	100%	100%	100%	100%	19
33℃	Control	100%	97%	87%	30%	23%	30
	Tg	100%	98%	98%	97%	68%	62
