# Supplementary figures and images for: Involvement of Heat Shock Proteins on the Transcriptional Regulation of Corticotropin-Releasing Hormone in Medaka
Source: Front Endocrinol (Lausanne). 2019 Aug 2;10:529. doi: 10.3389/fendo.2019.00529 (PMC6688511; doi:10.3389/fendo.2019.00529)

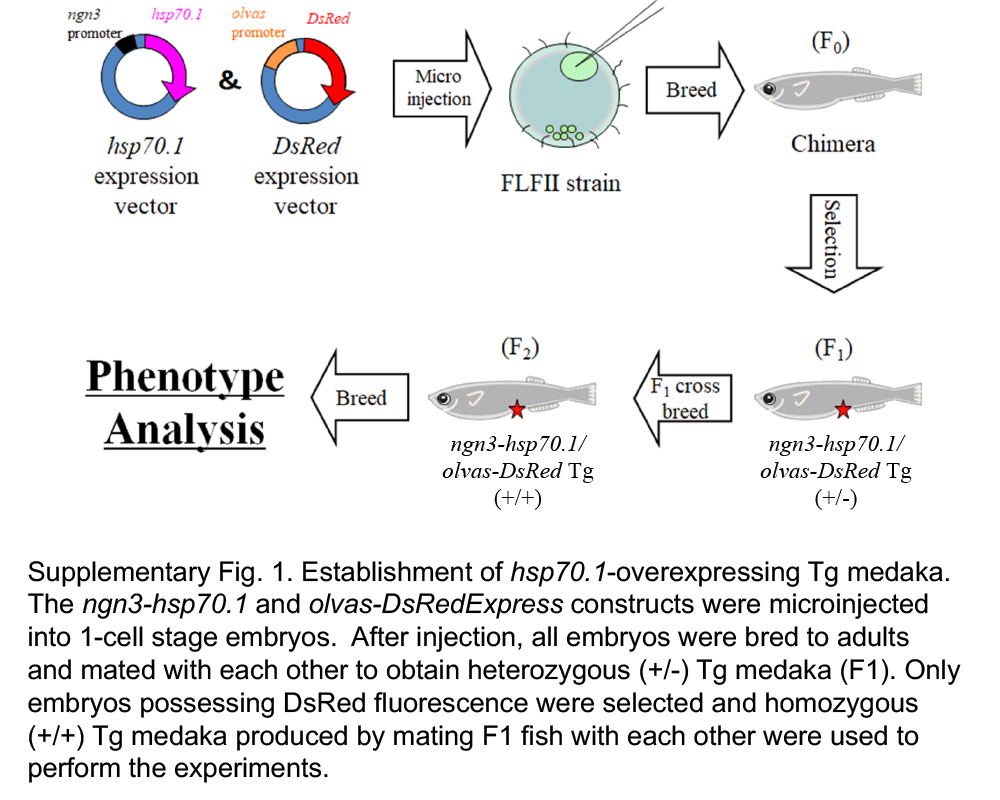

Supplement: Supplementary file 3 [file Image_1.tif]

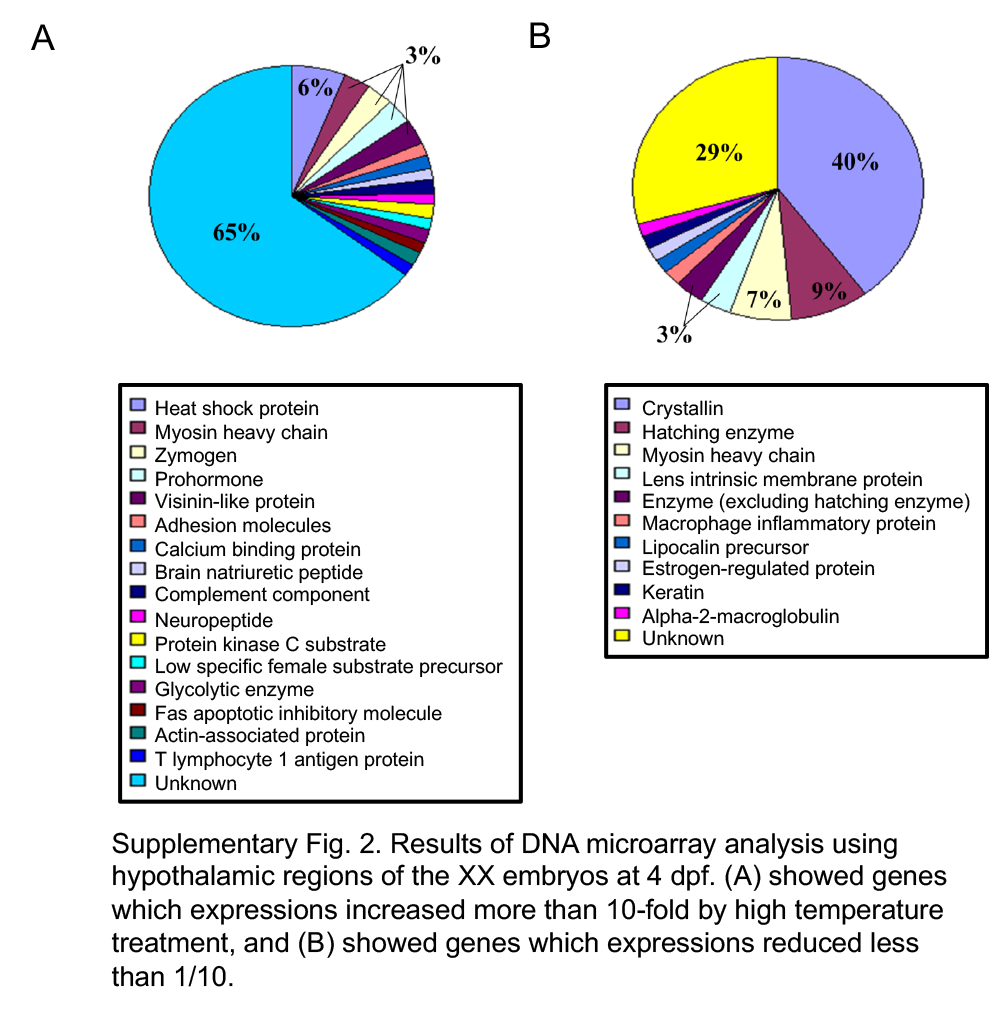

Supplement: Supplementary file 4 [file Image_2.tif]
